# Response to Isoniazid-Resistant Tuberculosis in Homeless Shelters, Georgia, USA, 2015–2017

**DOI:** 10.3201/eid2503.181678

**Published:** 2019-03

**Authors:** David P. Holland, Shanica Alexander, Udodirim Onwubiko, Neela D. Goswami, Aliya Yamin, Omar Mohamed, Rose-Marie Sales, Gail Grant, Phillip Talboy, Susan Ray, Kathleen E. Toomey

**Affiliations:** Fulton County Board of Health, Atlanta, Georgia, USA (D.P. Holland, S. Alexander, A. Yamin, O. Mohamed, K.E. Toomey);; Emory University, Atlanta (D.P. Holland, N.D. Goswami, S. Ray);; Centers for Disease Control and Prevention, Atlanta (S. Alexander, G. Grant, P. Talboy);; Georgia Department of Public Health, Atlanta (U. Onwubiko, R.-M. Sales, S. Ray)

**Keywords:** Tuberculosis, latent tuberculosis, homeless persons, homeless shelters, tuberculosis and other mycobacteria, Atlanta, Fulton County, Georgia, outbreak, antimicrobial resistance, isoniazid, TB

## Abstract

In 2008, an outbreak of isoniazid-resistant tuberculosis was identified among residents of homeless shelters in Atlanta, Georgia, USA. When initial control efforts involving standard targeted testing failed, a comprehensive approach that involved all providers of services for the homeless successfully interrupted the outbreak.

In 2008, the Tuberculosis Prevention and Control Program at the Georgia Department of Public Health (Atlanta, GA, USA) was notified of 7 cases of active tuberculosis (TB) in Fulton County, Georgia, for which isolate genotypes matched. All cases were linked to 1 homeless shelter in Atlanta, and all isolates exhibited low-level isoniazid resistance (resistant at 0.1 μg/mL but susceptible at 0.4 μg/mL according to BACTEC MGIT [Becton, Dickson and Company, https://www.bd.com]) (*1*). Additional cases with the same genotype were subsequently discovered at other locations. As of December 2017, a total of 110 outbreak-related cases had been identified in Fulton County (Figure 1), an additional 17 cases in other Georgia counties, and 47 cases in 15 other states. Of the 110 Fulton County cases, 41 (37%) patients were co-infected with HIV. A previous report of the outbreak through December 2015 identified several challenges associated with confronting the outbreak, notably lack of accurate rosters for overnight residents in most shelters, lack of voluntary participation in the episodic large-scale screenings (diagnostic testing with a tuberculin skin test [TST] or interferon-γ release assay, or a symptom screening for persons known to have a prior positive diagnostic test result) conducted at the shelters, and lack of consistent TB screening procedures at any of the facilities (*1*). An additional complication was ongoing litigation and efforts by nearby businesses and agencies of the Atlanta city government to close the index shelter (*2*). As a result of these issues, implementation of true targeted testing was initially limited, but in response to the large increase in the number of persons with a diagnosis of active TB caused by the outbreak strain in 2014 (Figure 1), the health department intensified its efforts. We report the results of the outbreak response in Fulton County, conducted from January 2015 through December 2017, illustrating several challenges caused by disjointed healthcare received by persons experiencing homelessness.

**Figure 1:**
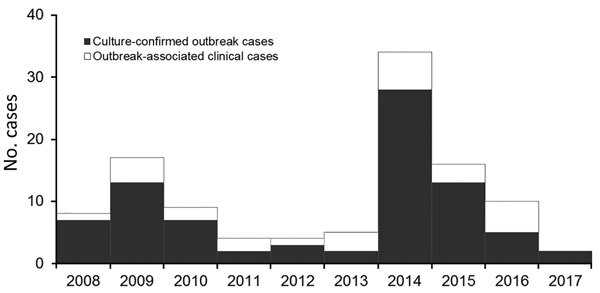
Outbreak-associated tuberculosis cases, Fulton County, Georgia, USA, 2008–2017. Culture-positive patients had >1 positive sputum culture with an isolate that had the outbreak-related genotype. Patients with clinical disease did not have a positive culture result but were epidemiologically linked to stays in homeless shelters before the diagnosis of tuberculosis was made.

## The Study

In June 2014, the Fulton County Board of Health joined with Mercy Care, a local federally qualified health center, to create the Metro Atlanta TB Task Force, including representatives from public health, homeless shelters, and other community service providers to provide a coordinated response in which testing targeted the entire population of persons experiencing homelessness instead of individual facilities. Before the task force interventions, none of the county’s 6 homeless shelters had adopted effective TB control guidelines (*1*). Furthermore, a formal survey of shelter employees revealed that their knowledge of TB disease and its transmission was low, a situation further exacerbated by rapid staff turnover. To address these issues, the task force began a series of meetings, led by the health department and Mercy Care, to develop and implement a set of comprehensive TB control guidelines for homeless shelters.

A key feature of these interventions was mandatory TB screening of shelter residents within 7 days of shelter entry and every 6 months thereafter. In addition, the guidelines included the following principles: appointment of a TB liaison at each of the 6 shelters, isolation or separation of residents with cough, maintenance of a bed log that includes every resident’s name and sleeping location within the facility, provision of a TB clearance card at the time each person completed screening to allow them access to the 6 shelters, and random inspections of the shelters by the health department to ensure adherence to the guidelines and to provide ongoing staff education. Because publication of the guidelines did not lead to their immediate uptake at all 6 shelters, educational initiatives were undertaken to increase TB knowledge and awareness of guideline content among shelter staff and the homeless community (*3*).

During the outbreak, persons with a diagnosis of latent TB infection were assumed to be infected with the isoniazid-resistant strain and were therefore offered daily treatment with rifampin for 4 months (*4*). Directly observed therapy was provided for most shelter residents starting in 2008 and for all residents after 2015; incentives ($5 coupons for local grocery stores) were offered weekly for successful completion of all doses.

Data from testing performed before 2015 showed that ≈30% of persons tested by TST did not return for reading of the results. To overcome this problem, starting in 2015, the TST was replaced as the primary test by a single-step blood test, an interferon γ-release assay (QuantiFERON Gold In-Tube; QIAGEN, https://www.qiagen.com).

**Figure 2:**
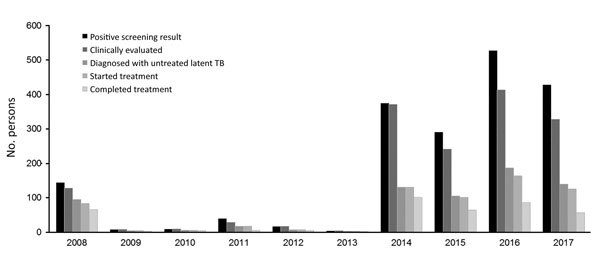
Cascade of care for persons experiencing homelessness who were tested for latent tuberculosis (TB) infection, Fulton County, Georgia, USA, 2008–2017. Positive screening result indicates number with either a new or prior positive test result at screening; clinically evaluated indicates number evaluated by a clinician; diagnosis of untreated latent TB indicates number with a diagnosis of latent TB who required treatment; started treatment indicates number who started treatment for latent TB; and completed treatment indicates number who completed treatment.

From January 2015 through December 2017, a total of 14,496 persons were screened for latent TB infection, compared with 2,451 from January 2008 through December 2014 (Figure 2). During 2015–2017, a total of 3 cases of active TB were identified through screening, resulting in disease incidence of 20.7 cases/100,000 persons screened. From January 2, 2015, through December 31, 2017, a total of 430 persons received a diagnosis of untreated latent TB and 391 (91%) started treatment; of those starting treatment, 207 (53%) completed a full course. In 2017, only 2 cases of outbreak-associated TB were reported (Figure 1).

## Conclusions

Despite an ongoing downward trend of TB in the United States (*5*), outbreaks among persons with a history of homelessness continue to occur regularly (*6*–*12*). As previously reported, the mobility of persons experiencing homelessness rendered standard contact tracing activities in this investigation difficult (*13*). Greater success was achieved with a systematic approach targeting all shelter residents for testing rather than just individual facilities in which cases had been identified. In particular, the number of persons tested and provided treatment for latent TB markedly increased after introduction of comprehensive standard screening guidelines and implementation at all shelters.

Since 2015, testing for and diagnosis of latent TB increased despite a decrease in outbreak-associated active TB cases. Acceptance of latent TB treatment was >90%, which in our estimation was attributable to the universal support of the shelter providers. Regular inspections of facilities by public health staff provided frequent opportunities for ongoing education about TB and the need to maintain vigilance despite the downward trend in case numbers.

The primary challenge that remains is finding the substantial numbers of persons with untreated latent TB who are at risk for active TB but no longer in the shelter system. Because of this population, we anticipate that episodic cases of active TB with the outbreak genotype will occur. However, if the comprehensive guidelines now in place are properly implemented, reemergence of TB with the outbreak strain among shelter residents, as was seen in 2014, can be prevented. In addition, efforts are under way to increase the identification of persons with HIV (either not diagnosed or in persons not receiving care) and facilitate the rapid initiation of antiretroviral therapy along with the supportive measures required to maintain treatment adherence.

The presence of isoniazid resistance in the outbreak strain complicated treatment of active disease and latent infection (*4*,*14*). Unfortunately, 1 person with isoniazid-resistant TB diagnosed in 2008 later died of multidrug-resistant TB (*15*).

Homelessness presents several challenges to caring for persons with active TB and those who have been exposed to TB (https://www.cdc.gov/tb/topic/populations/homelessness/default.htm). Congregate living in shelters can provide an efficient means of transmission. Once tested, persons frequently move into, out of, and within shelter systems, making follow-up difficult. Also, shelter residents frequently lack a permanent place to store medications, which necessitates the use of directly observed therapy. In the outbreak we report, these challenges were overcome with a systematic operational approach focused on the entire population of persons experiencing homelessness, illustrating that such approaches can contribute to effective control of TB outbreaks within these populations.
